# Production losses attributable to suicide deaths in European Union

**DOI:** 10.1186/s12889-021-11010-5

**Published:** 2021-05-19

**Authors:** Błażej Łyszczarz

**Affiliations:** Department of Health Economics, Faculty of Health Sciences, Nicolaus Copernicus University in Toruń, ul. Sandomierska 16, 85-830 Bydgoszcz, Poland

**Keywords:** Suicides, Production losses, Indirect costs, European Union

## Abstract

**Background:**

Suicide is an important public health problem with multidimensional consequences for societies. One of the under-researched areas of suicide consequences are cross-country analyses of production losses associated with these deaths. The aim of this study was to estimate the production losses (indirect cost) of suicide deaths in 28 European Union states (EU-28) in 2015.

**Methods:**

The study used societal perspective and human capital approach to investigate production losses due to suicide mortality at working age. Eurostat’s data on the number of deaths was used to identify suicide mortality burden in terms of years of potential productive life lost. Labour and economic indicators were applied to proxy the discounted value of potential economic output lost. A one-way deterministic sensitivity analysis was conducted to test the robustness of the estimates.

**Results:**

The production losses attributable to suicide deaths in EU-28 in 2015 were €9.07 billion. The per suicide indirect cost of these deaths was €231,088 for the whole EU-28 population; Luxembourg experienced the highest per suicide burden of €649,148. The per capita production losses of suicides in EU-28 was €17.80 and Ireland experienced the highest per capita burden of €48.57. The losses constituted an economic burden of 0.061% of EU-28’s GDP and this share ranged from 0.018% in Cyprus to 0.161% in Latvia. Most of the losses (71–91%) were due to men’s deaths. The results of the sensitivity analysis exhibit a large variation of losses; the highest (lowest) cost was identified with no adjustment for lower employment rates among those dying by suicide (adjustment for minimum productivity) and was 92.3% higher (59.7% lower) on average than in the base scenario.

**Conclusion:**

Public health actions aimed at prevention of suicides might reduce their health burden but also contribute to the economic welfare of European societies.

**Supplementary Information:**

The online version contains supplementary material available at 10.1186/s12889-021-11010-5.

## Background

Suicide, being in the top twenty causes of death worldwide, is considered to be a global health problem. Around 800 thousand people die from suicide each year [1] and over 56,000 of 5.2 million deaths in the European Union (EU) in 2015 were due to intentional self-harm [2]. The consequences of suicide are numerous; for a typical death by suicide, at least six people are directly affected in terms of human suffering [3]. Moreover, major economic consequences, including increased medical utilization, time absent from work, and production output lost are experienced by several groups [3, 4].

Previous contributions aiming at assessing the cost of suicides, include studies from the United States [5–8], Poland [9], Ireland [10], Australia [4, 11] and Spain [12] among others. However, this topic seems to be understudied; so far no systematic reviews have been published and the only cross-country study on the cost of suicide is concerned with the youth deaths in highly developed countries [13]. International comparisons of suicides’ cost are difficult because particular studies use different methodological approaches, data sources and encompass various cost components. The most comprehensive approaches to the economic burden of suicide include estimation of following cost categories: medical care costs (direct costs), autopsy and investigation costs (medicolegal costs), production losses (indirect costs) and cost of pain and stress (intangible costs). Such studies provide an overall picture of suicides’ cost but are only feasible in a national context and require exhaustive and reliable data sources. This research aims to overcome the low comparability of previous findings from different countries by estimating a single cost category of production losses associated with suicide mortality across the 28 EU countries. With the use of uniform data from all the EU states, the study provides highly comparable estimates of the European states’ economic burden of suicide. Although, suicide mortality is only a single cost category, several studies show that it is a crucial one which constitutes a majority of economic burden of suicide with shares of total economic cost as large as 91–98% in the United States [5–7], 94% among Australian youth [4] and 84% in New Zealand [14]. Moreover, this single cost category has also been subject to previous studies [9, 12] concerned with the production losses attributable to completed suicides solely.

Therefore, the aim of this study was to estimate the production losses (indirect cost) associated with suicide mortality among population at working age in the 28 EU countries in the year 2015. Understanding the magnitude of this economic burden is important for assessing potential savings from cost-effective suicide prevention programmes. With introduction of such programmes, the number of suicide deaths could be reduced bringing benefits in terms of not only lives saved and trauma avoided but also economic output gained. Moreover, the estimates provided here might be useful to compare the economic burden of various health problems and, as such, to facilitate prioritization of health policy choices both nationally and at a supranational level.

## Methods

The methodological approach used in this research builds on a model designed to estimate the production losses of premature mortality [15] which also has recently been applied for assessment of indirect cost associated with alcohol-attributable mortality [16] in EU countries.

### General modelling approach

The study used population-based data, the societal perspective [17, 18] and human capital approach (HCA) [19, 20] to estimate the production losses attributable to premature mortality associated with suicides in the 28 EU countries. Premature mortality is defined as those cases of death that occur at working age. The study only accounts for losses borne in formal economy while it does not encompass the value of informal activities foregone due to suicide deaths. With the use of HCA, the indirect cost of mortality was proxied by the discounted value of economic output that would be produced if those who died prematurely were still alive and working until the average age of exiting labour market [21, 22]. Per worker gross domestic product (GDP) was used to measure productivity and the estimates were adjusted for the decreasing marginal productivity of labour by applying a 0.65 adjustment coefficient. Generally, the use of marginal productivity is a preferred choice over average productivity in indirect cost estimation [23, 24] and, therefore, this coefficient was used to account for the law of diminishing marginal productivity. This adjustment results from the fact that the production process relies on several inputs and diminishing one of them (labour) affects only a respective proportion of the production output [25, 26]. Hence, the production lost due to suicide deaths should not be considered to be as high as the average output. The value of 0.65 used reflects the proportion of output attributable to labour by applying a relationship between marginal and average labour productivity; therefore, this adjustment reflects output elasticity of labour in Cobb-Douglas production function as used in the European context [27].

Unless stated otherwise, all the data refer to the year 2015.

### Data sources

The age- and gender-specific data on the number of suicides was taken from Eurostat’s dataset of death causes which reports figures on intentional self-harm mortality [28]. The country-specific data on labour market characteristics used to determine the duration of working life was taken from European Commission’s report on aging [29] (the average effective age of exit from labour market), Eurostat’s database [30] (the employment rates) and provided by Eurostat on the author’s request (the average age of starting first regular job). The population and economic output data was taken from Eurostat [31], while the future country-specific potential per worker GDP growth rates from report [29]. The lower employment rates in the suicide population were proxied by the country-specific data on employment among those with chronic depression [32], which was the only available measure to proxy employment in suicide population for a range of EU countries.

### Estimation strategy

In the first stage, the age- and sex-specific number of suicide deaths at 5-years age intervals (0–4; 5–9; …; 60–64; 65–69 years) was extracted from Eurostat database. In order to identify the production loss at a particular age, it was necessary to identify the number of deaths at this certain age. It could be done by assuming that the distribution of deaths in particular intervals was even.^1^ The average number of suicide deaths throughout the 3-year period of 2014–2016 was used in order to remove the effect of unusual variation in mortality, particularly observable in low populated countries [15]. A half-cycle adjustment was applied meaning that all deaths occurred in the middle of the year [33]. The measure of years of potential productive life lost (YPPLL) [9, 34] was used to weight suicide cases occurring at various ages.

The second stage was to identify the mean time a person at each age would work if had not died from suicide; the following country- and sex-specific labour market measures were used for this purpose: the average age of starting first regular job^2^; the average age of exiting the labour market (data for the year 2016) [29]; and (5-years) age-specific employment rates^3^ [30]. These measures were assumed to be unchanged in future, because of the uncertainty of labour market situation in the years to come. Using these labour measures, the average time of work lost due to suicide death was identified separately for men and women at every working age for each of the states.

The average production lost due to suicide was proxied by per worker GDP adjusted for purchasing power parity (PPP)^4^ and marginal productivity coefficient (0.65). However, to reflect the fact that those dying from suicide are less likely to work, the employment rates of people reporting chronic depression [32] were used and these ranged from 29.5 to 73.0% of employment rates of those without depression in Cyprus and Germany, respectively. The reasoning for using this measure was based on the fact that at least 90% people who die from suicide have suffered from mental disorders with depression being a major one [35]; moreover, previous studies allocate 50–70% of suicides to those who have suffered from depression [36–38]. However, this choice seems to be conservative comparing to the previous studies which assume much lower employment differences between general and suicide populations, e.g. a 2.25% employment rate gap in the Irish [10] and Scottish [39] studies.

The future losses were discounted using a 5% rate and country-specific potential per worker GDP growth rates for each forthcoming decade [29] were applied to reflect the growth of the EU economies.

**Fig. 1:**
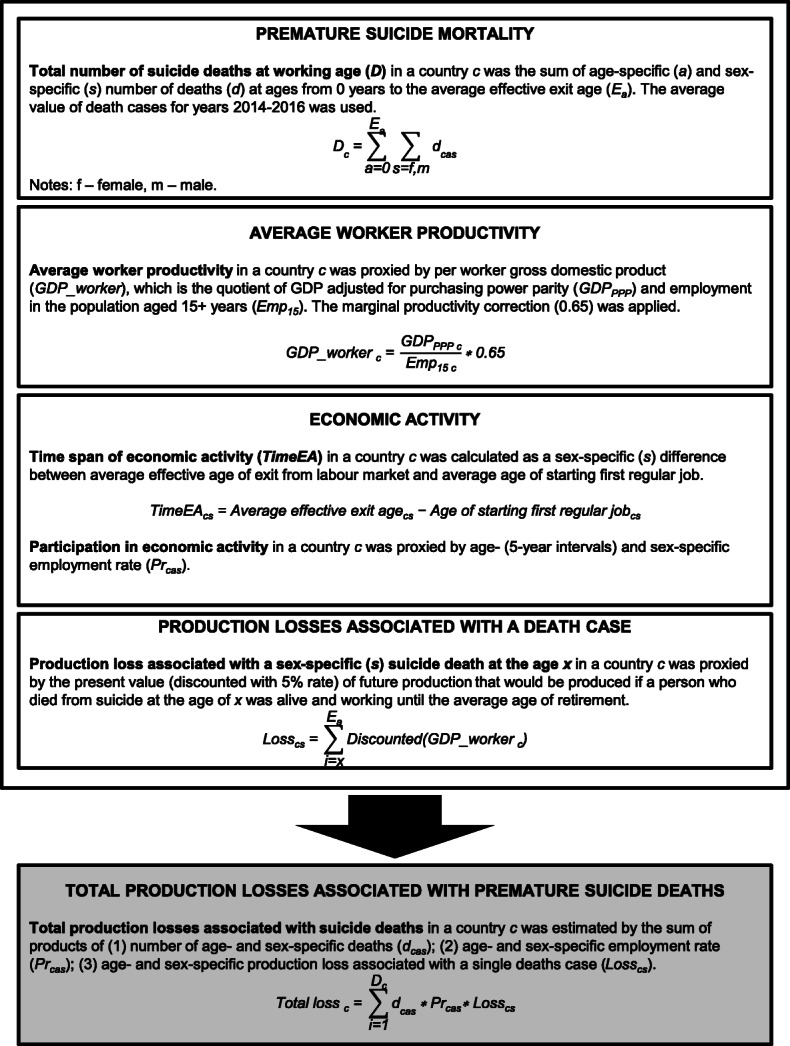
Formulas for calculating production losses associated with suicide deaths in European Union

The formulas used for calculating the production losses are depicted in Fig. 1.

### Sensitivity analysis

A one-way deterministic sensitivity analysis^5^ was performed to test the magnitude of changes in the model’s parameters. The following sensitivity scenarios were used: a 3.5 and 0% discount rate (DR); a ± 0.05 variation of the 0.65 correction coefficient; gross value added^6^ (GVA) instead of GDP and 0 and 2% future economic growth for all the countries. To deal with uncertainty in the future labour market situation two scenarios using homogeneous data for all the states were assumed: average EU values of labour measures; and ages 18 and 67 used as the ages of labour market entry and exit, respectively [15]. Also, scenarios were tested in which all deaths occurred among minimally productive workers^7^; and with no depression-specific labour employment rate adjustment. Eventually, all the deaths (for those aged 10+ years of age) reported as events of undetermined intent were added to suicide cases to make an attempt to account for under-reporting of suicides. The other possible scenario to be included into sensitivity analysis would be to differentiate male and female productivity. Unfortunately, no sex-specific data on average GDP produced is available and the only figure broken down by sex which could be potentially useful was gender pay gap. This last measure is considered to reflect rather labour market discrimination [40, 41] than real productivity differences; thus, it was not included as a sensitivity scenario.

## Results

### Suicide mortality at working age and YPPLL

There were 39,265 (estimated) deaths at working age due to suicide in the European Union in 2015 (Table 1). The number of deaths was highest in the Western Europe (WE) countries (16,085 cases) followed by the Central and Eastern Europe (CEE) (9010), Northern Europe (NE) (7315) and Southern Europe (SE) (6854) countries. A majority of the premature deaths was associated with men’s suicides (from 75.3% in WE countries to 86.2% in CEE countries). The suicide rate for the whole EU community was 7.7 cases per 100,000 population and 3.2 (12.4) for women (men). The overall suicide rate was highest in the CEE states (9.9 cases per 100,000 population) and lowest in SE (5.0). Men’s suicide rate was disproportionally high in CEE (17.6) where it was more than twice of the rate in SE (7.9). On the other hand, the rate for women was more even in the sub-regions with the highest values in WE (4.2) and NE (3.3). The countries with the greatest number of suicides at working age were Germany (6438), France (5863) and Poland (4433) while there were less than 40 cases of such deaths in Cyprus, Luxembourg and Malta. However, the relative burden of suicide deaths was highest in Lithuania (23.4 cases per 100,000 pop.), Latvia (13.9) and Belgium (12.4) while the rate of suicides was lowest in Greece (3.3 cases), Cyprus (4.1) and Italy (4.3).

**Table 1:** Number and incidence of deaths at working age and years of potential productive life lost due to suicide mortality in European Union countries in 2015

	Number of suicide deaths^a^			Suicide deaths^a^ per 100,000 pop.			YPPLL			YPPLL per 10,000 pop.		
	Men	Women	Total	Men	Women	Total	Men	Women	Total	Men	Women	Total
**Central and Eastern Europe**	**7764**	**1246**	**9010**	**17.6**	**2.7**	**9.9**	**150,126**	**21,082**	**171,208**	**34.1**	**4.5**	**18.9**
Bulgaria	325	78	403	9.3	2.1	5.6	5968	1322	7290	17.1	3.6	10.2
Czechia	858	176	1034	16.6	3.3	9.8	17,265	3206	20,470	33.3	6.0	19.4
Hungary	941	245	1187	20.1	4.8	12.1	15,558	3559	19,117	33.2	6.9	19.4
Poland	3932	501	4433	21.4	2.6	11.7	79,802	8844	88,645	43.4	4.5	23.3
Romania	1400	194	1595	14.5	1.9	8.1	25,986	3323	29,309	27.0	3.3	14.8
Slovakia	308	52	360	11.6	1.9	6.6	5548	828	6377	20.9	3.0	11.7
**Northern Europe**	**5697**	**1619**	**7315**	**11.9**	**3.3**	**7.5**	**125,570**	**34,393**	**159,963**	**26.1**	**7.0**	**16.4**
Denmark	329	107	435	11.6	3.7	7.7	6322	1954	8276	22.4	6.8	14.6
Estonia	131	25	156	21.2	3.6	11.9	2943	534	3476	47.7	7.6	26.4
Finland	467	135	602	17.2	4.9	11.0	10,307	2988	13,294	38.1	10.7	24.2
Ireland	327	80	407	13.9	3.4	8.6	8005	1929	9934	34.0	8.1	21.0
Latvia	229	44	273	25.5	4.1	13.9	4584	815	5400	50.9	7.6	27.4
Lithuania	588	87	675	44.5	5.6	23.4	12,083	1575	13,658	91.5	10.1	47.3
Sweden	613	259	873	12.3	5.3	8.8	13,589	5723	19,312	27.3	11.7	19.6
United Kingdom	3012	881	3893	9.3	2.7	6.0	67,736	18,875	86,612	20.9	5.7	13.2
**Southern Europe**	**5251**	**1603**	**6854**	**7.9**	**2.3**	**5.0**	**96,498**	**28,558**	**125,057**	**14.6**	**4.1**	**9.2**
Croatia	335	94	429	16.6	4.3	10.2	5678	1387	7065	28.2	6.4	16.9
Cyprus	27	8	35	6.6	1.8	4.1	648	172	820	15.6	3.9	9.6
Greece	284	72	357	5.4	1.3	3.3	4937	1240	6178	9.5	2.2	5.7
Italy	2039	580	2619	6.9	1.9	4.3	38,189	10,227	48,416	13.0	3.3	8.0
Malta	23	6	29	10.1	2.9	6.5	469	132	601	20.5	5.9	13.3
Portugal	489	163	653	10.0	3.0	6.3	8690	2701	11,391	17.8	5.0	11.0
Slovenia	204	38	242	19.9	3.7	11.7	4034	677	4711	39.4	6.5	22.8
Spain	1849	641	2490	8.1	2.7	5.4	33,853	12,022	45,876	14.8	5.1	9.9
**Western Europe**	**12,110**	**3975**	**16,085**	**13.3**	**4.2**	**8.7**	**221,667**	**67,125**	**288,792**	**24.3**	**7.1**	**15.5**
Austria	624	184	807	14.5	4.2	9.3	12,286	3181	15,467	28.6	7.2	17.8
Belgium	1003	391	1395	18.1	6.8	12.4	18,353	6136	24,489	33.1	10.7	21.7
France	4506	1358	5863	13.9	4.0	8.8	77,555	21,826	99,382	24.0	6.4	14.9
Germany	4863	1574	6438	12.1	3.8	7.9	91,642	27,562	119,203	22.8	6.6	14.6
Luxembourg	31	8	39	10.6	2.9	6.8	520	131	651	17.8	4.6	11.3
Netherlands	1083	460	1543	12.8	5.4	9.1	21,310	8290	29,600	25.2	9.7	17.4
**European Union**	**30,821**	**8444**	**39,265**	**12.4**	**3.2**	**7.7**	**593,862**	**151,158**	**745,020**	**23.8**	**5.8**	**14.6**

Notes: The mortality figures refer to average 2014–2016 values for each country. a – deaths at country- and sex-specific working age; *YPPLL* years of potential productive life lost

Suicide mortality led to 745,020 YPPLL in the EU and this number was highest in the WE states (288,792) followed by the CEE (171,208), NE (159,963) and SE countries (125,057). The number of YPPLL was three to seven times higher for men’s suicides compared to women’s and the difference between the sexes in this respect was most pronounced in CEE. The YPPLL rate was 14.6 per 10,000 population in the whole EU (5.8 for women and 23.8 for men) and ranged from 9.2 in SE to 18.9 in CEE. The rates of YPPLL associated with men’s suicides were much higher than those of women’s in all the sub-regions. The YPPLL rate (per 10,000 pop.) was highest in three Baltic states – Lithuania (47.3), Latvia (27.4) and Estonia (26.4) – while it was lower than ten in four SE countries, namely Greece (5.7), Italy (8.0), Cyprus (9.6) and Spain (9.9).

### Production losses associated with suicide mortality

The total production losses (indirect cost) associated with suicide mortality at working age in 2015 were €9.07 billion in the EU and almost half of this burden was observed in the WE countries (€4.50 billion). The cost in other sub-regions was €1.16 billion in SE, €1.43 billion in CEE and €1.98 billion in NE (Table 2). A vast majority of this cost was due to male mortality (from 78.8% in WE to 89.0% in CEE). The indirect cost was highest in Germany (€2.04 billion), France (€1.60 billion) and the United Kingdom (€1.00 billion) while it was less than €10 million in Cyprus (€3.6 million) and Malta (€5.5 million). In relative terms, the economic burden of suicide mortality in the whole EU economy was 0.061% of GDP and ranged from 0.033% in SE countries to 0.081% in CEE. The highest share of GDP lost was estimated for three Baltic states – Latvia (0.161%), Lithuania (0.143%) and Estonia (0.124%) – while this burden was lowest in Cyprus (0.018%), Greece (0.022%) and Italy (0.030%).

**Table 2:** Production losses (indirect cost) attributable to suicide mortality in European Union countries in 2015

	Total cost (€ 1000 PPP)			Total cost as % GDP	Cost per capita			Cost per suicide		
	Men	Women	Total		Men	Women	Total	Men	Women	Total
**Central and Eastern Europe**	**1,272,742**	**157,807**	**1,430,549**	**0.081**	**14.03**	**1.74**	**15.77**	**163,930**	**126,602**	**158,766**
Bulgaria	30,311	6396	36,707	0.037	4.22	0.89	5.11	93,347	82,053	91,160
Czechia	210,792	35,234	246,026	0.092	19.99	3.34	23.33	245,631	200,526	237,966
Hungary	104,058	22,179	126,237	0.065	10.58	2.26	12.84	110,555	90,402	106,388
Poland	736,863	73,173	810,036	0.106	19.40	1.93	21.33	187,417	146,061	182,743
Romania	143,331	14,449	157,780	0.049	7.25	0.73	7.98	102,355	74,410	98,952
Slovakia	47,386	6376	53,762	0.044	8.73	1.17	9.90	153,933	121,844	149,271
**Northern Europe**	**1,579,419**	**402,398**	**1,981,816**	**0.063**	**16.23**	**4.13**	**20.36**	**277,256**	**248,618**	**270,920**
Denmark	89,916	24,941	114,857	0.055	15.82	4.39	20.21	273,578	233,470	263,739
Estonia	30,140	5325	35,465	0.124	22.89	4.04	26.93	230,357	210,760	227,185
Finland	132,008	37,488	169,496	0.097	24.05	6.83	30.88	282,815	276,950	281,496
Ireland	191,894	37,805	229,699	0.094	40.58	7.99	48.57	586,116	474,104	564,178
Latvia	50,566	8420	58,986	0.161	25.67	4.27	29.95	220,356	191,512	215,718
Lithuania	79,697	10,674	90,372	0.143	27.60	3.70	31.29	135,519	122,562	133,848
Sweden	202,644	82,750	285,394	0.081	20.53	8.38	28.92	330,376	319,245	327,070
United Kingdom	802,553	194,994	997,546	0.049	12.27	2.98	15.26	266,452	221,312	256,236
**Southern Europe**	**947,177**	**215,449**	**1,162,626**	**0.033**	**6.97**	**1.59**	**8.56**	**180,388**	**134,367**	**169,622**
Croatia	27,748	6244	33,991	0.047	6.62	1.49	8.11	82,911	66,242	79,248
Cyprus	2957	672	3628	0.018	3.48	0.79	4.27	108,568	87,620	103,966
Greece	39,934	7142	47,076	0.022	3.70	0.66	4.36	140,502	98,683	132,015
Italy	418,556	80,014	498,571	0.030	6.90	1.32	8.21	205,246	137,924	190,336
Malta	4698	779	5477	0.046	10.43	1.73	12.15	203,093	122,953	185,868
Portugal	80,495	21,919	102,414	0.044	7.79	2.12	9.91	164,450	134,355	156,927
Slovenia	38,999	6068	45,067	0.092	18.89	2.94	21.82	191,142	158,952	186,069
Spain	333,790	92,612	426,402	0.035	7.19	1.99	9.18	180,552	144,398	171,240
**Western Europe**	**3,547,092**	**951,627**	**4,498,719**	**0.071**	**19.08**	**5.12**	**24.19**	**292,907**	**239,377**	**279,677**
Austria	151,293	36,919	188,211	0.058	17.39	4.24	21.64	242,586	200,863	233,089
Belgium	228,860	69,342	298,202	0.077	20.30	6.15	26.45	228,085	177,194	213,806
France	1,278,819	323,312	1,602,131	0.079	19.17	4.85	24.01	283,817	238,159	273,246
Germany	1,606,692	432,078	2,038,771	0.069	19.67	5.29	24.96	330,361	274,452	316,689
Luxembourg	20,963	4431	25,395	0.058	36.38	7.69	44.07	677,983	540,423	649,148
Netherlands	260,465	85,545	346,010	0.055	15.33	5.03	20.36	240,563	185,876	224,251
**European Union**	**7,346,429**	**1,727,281**	**9,073,710**	**0.061**	**14.41**	**3.39**	**17.80**	**238,356**	**204,560**	**231,088**

The indirect cost of suicide mortality per capita in 2015 was €17.80 in the EU (€14.41 for male and €3.39 for female suicides). This average cost was highest in WE (€24.19) and NE (€20.36), followed by the CEE (€15.77) and SE (€8.56) states. Ireland experienced the greatest per capita economic burden in absolute values with the indirect cost of €48.57 per person and other countries with large average losses were Luxembourg (€44.07) and Finland (€30.88). Finally, the average cost per suicide was €231,088 in the EU and varied sub-regionally from €158,766 in CEE to €279,677 in WE. This per suicide cost was highest in Luxembourg (€649,148), Ireland (€564,178) and Sweden (€327,070) while the lowest values were observed in Croatia (€79,248), Bulgaria (€91,160) and Romania (€98,952). For the whole EU community, the indirect cost of a single male suicide was 16.5% higher than a female suicide.

**Fig. 2:**
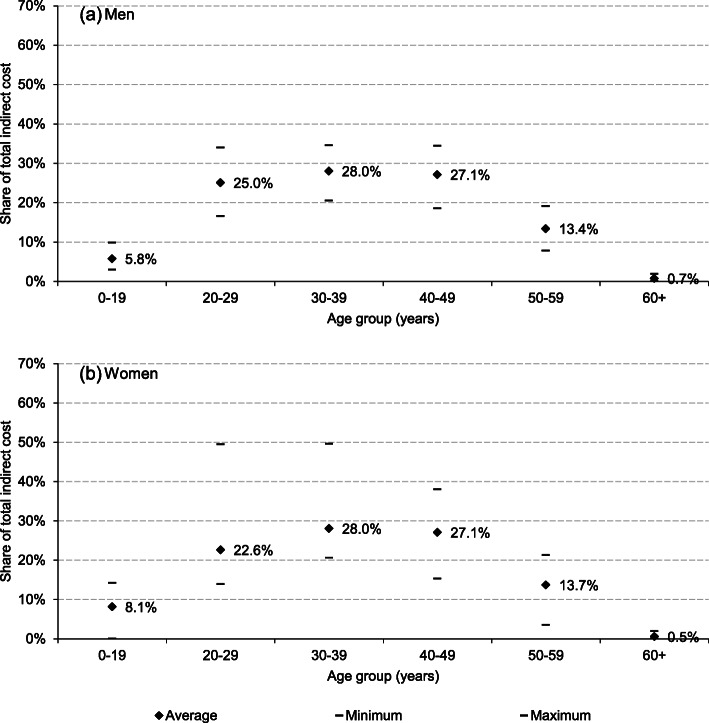
Age groups distribution of production losses attributable to suicide mortality in European Union countries in 2015. Notes: Average shares are calculated as unweighted means

In the whole group of countries, the highest share of mortality cost was associated with suicides of those at ages 30–39 and 40–49 years (Fig. 2; country-specific results in Additional file 1). A slightly lower share of total losses was due to young adults’ suicides (20–29 years) and this share was higher for men than women. On the other hand, the relative magnitude of losses attributable to mortality among those being between 0 and 19 years of age was higher for women. Additionaly, the between-country variation in all these shares was much higher for women than men.

### Sensitivity analysis

The one-way deterministic sensitivity analysis shows that the cost estimates are subject to notable variation as a result of changes in some of the model’s parameters (Table 3). The high changes in the cost result from the scenario which does not account for decreased employment rate in suicide population; the production losses would be 92.3% higher on average if general population employment rates were applied (from 36.9% in Germany to 238.9% in Cyprus). On the other hand, the cost would be 59.7% lower on average if minimum productivity adjustment was used. Using the 3.5% discount rate raised the cost by 17.4% on average, with relatively low variation among countries (14.9–20.5%). With no discounting, the indirect cost would be 86.5% higher on average. Using GVA instead of GDP as a productivity measure decreased the estimates by 11.6% on average. The assumption of 0% (2%) future economic growth changed the results by −13.4% (3.8%) on average. The use of alternative data on labour market characteristics had minor effect on the losses estimated; with the entry age of 18 and exit age of 67 for both sexes the results changed by 5.5% on average with the highest (lowest) change of 14.9% (1.8%) in Luxembourg (Sweden). When the average EU values for these labour measures were used for all the countries, the cost variation was even lower. Classification of all undetermined deaths as suicides had varying effect on estimates and this depended on the country; for Slovakia, the cost was as much as 77.4% elevated while for Italy it was only 0.1% higher. A detailed, country-specific sensitivity analysis is available online in Additional file 2.

**Table 3:** Summary of sensitivity analysis for estimates of production losses attributable to suicide deaths in European Union countries in 2015

	Average^a^ change	Minimum change from base scenario	Maximum change from base scenario
Discount rate (BS: 5%)			
3.5%	17.4%	14.9% (Greece)	20.5% (Latvia)
0%	86.5%	71.1% (Greece)	105.7% (Estonia)
Coefficient to adjust for decreasing marginal labour productivity (BS: 0.65)			
0.6	−7.7%	−7.7% (all countries)	
0.7	7.7%	7.7% (all countries)	
Productivity measure (BS: gross domestic product)			
Gross value added	−11.6%	−7.4% (Ireland)	−16.0% (Croatia)
Minimum productivity adjustment^b^ (BS: average productivity)			
	−59.7%	−47.6% (Slovenia)	−65.7% (Spain)
No lower employment rate adjustment (BS: employment rate for those reporting chronic depression)			
	92.3%	36.9% (Germany)	238.9% (Cyprus)
Future economic growth (BS: country-specific)			
0% for all the countries	−13.4%	−2.0% (Italy)	−33.5% (Latvia)
2% for all the countries	3.8%	0.1% (Hungary)	−19.9% (Latvia)
Labour market entry and retirement age (BS: country-specific)			
Sex–specific, average EU values	−0.4%	0.2% (Czechia)	8.1% (Luxembourg)
Both sexes: 18 years and 67 years	5.5%	1.8% (Sweden)	14.9% (Luxembourg)
Events of undetermined deaths included	19.2%	0.1% (Italy)	77.4% (Slovakia)

Notes: a – unweighted average; b – minimal productivity was obtained by dividing the minimum wage by the average wage in particular economies. There was no minimum wage legislation in six EU countries in 2015 (Denmark, Italy, Cyprus, Austria, Finland, Sweden); therefore, the weighted (by population) average value for the remaining countries was used for the states with no minimum wage; this mean was 39.3% of the average wage. Data for undetermined deaths in Cyprus was not available for none of the years; for some other countries and age intervals only some data was obtainable

## Discussion

This is the first study which aims to identify the cross-country economic burden of suicide in general population by estimating the production losses associated with suicide deaths in 28 EU countries. By applying a HCA model and Eurostat data, I obtained highly comparable estimates which reflect the specificity of economic burden experienced in particular countries.

The total production losses attributable to suicides at working age in the EU in 2015 were €9.07 billion, being a result of 39,265 suicides and 745 thousand years of potential productive life lost. A notable geographical difference was observed among the member states both in terms of the suicide rates and cost itself. All in all, the countries experiencing the highest relative burden of suicides were three Baltic states (Estonia, Lithuania, Latvia), Poland and Finland. On the other hand, the ones with the lowest detrimental economic effect of suicides were Cyprus, Greece, Italy, Spain and Bulgaria. Therefore, the countries located in the southern regions of Europe experienced minor health and economic burden of suicide compared to those situated in the north. This clearly reflects the geographical distribution of suicide mortality, particularly low death rates in the Mediterranean states and higher rates in Scandinavia and the Baltic post-Soviet republics. However, this mortality pattern is strengthened in terms of production losses because the NE states generally have higher employment rates (Denmark, Sweden, Latvia) and/or longer time of economic activity (Estonia, Ireland, Sweden) and/or higher prospects of future economic growth (Lithuania, Latvia, Estonia) than the SE countries. On the other hand, the greatest relative economic burden, measured by the share of GDP lost, in the CEE countries arises mainly from high suicide rates among men and dynamic projected economic growth which make future losses greater.

The results show that the cost of suicide is mainly driven by males’ death and this obviously reflects the sex disparities in suicide incidence [42, 43]. As much as 89.0% of production losses were due to suicides of men in CEE and this share was 71.0% in Sweden, a country with the most even gender distribution of cost. Considering the age distribution of suicide cost, the highest burden was identified for those at the ages 30–39 and 40–49 years. The age distribution of losses was similar for both sexes with a slightly higher magnitude of cost shares among women aged 0–19 years and young men (20–29 years).

The stability of results was tested using a one-way deterministic sensitivity analysis. The results changed notably with no discounting applied and this can be explained by the fact that suicide mortality is disproportionally high among relatively young people. Another scenario which varied estimates markedly was the use of general population employment rates instead of rates for depression population. It should be emphasized that this choice regarding employment rate is crucial for the indirect cost estimates. Yet, I decided to opt for the conservative scenario of a heavily reduced employment rate because the previous adjustment in this respect might be understated. For most other parameter changes used in the sensitivity analysis, the results’ variation was lower.

Because this is the first cross-country study on the cost of suicide mortality in general population, the findings of this research could only be compared to previous estimates from single states. The recent results from Spain [12] identified the production losses of €380.5 million in 2013 (in 5% DR scenario) which is a close value to my estimate of €426.4 million in 2015. This similarity is irrespective of some important differences in the methods and data sources used; e.g. for the productivity measure, the Spanish study applied gross salary while mine–GDP adjusted with 0.65 coefficient. The study from Poland [9] reported the indirect cost of completed suicides as equivalent to €694.5 million in 2012 (using 5% DR) and this value is, again, comparable to my estimate of €810.0 million in 2015 for this country. This similarity might be surprising regarding the fact that my estimates are adjusted for lower employment rates among those who killed themselves. According to the conservative adjustment used here, the employment rates in the populations under study are 33.2 percentage points lower in Spain and 31.7 percentage points lower in Poland as compared to general employment rates. Therefore, this similarity of results seems to be coincidental. However, I argue that using employment rates for general population in a country bias the results upward because those dying by suicide are possibly less likely to work. Finally, the Irish study identified the cost of lost market output attributable to suicides in 2001 and 2002 to be €205.9 million and €192.8 million, respectively [10]. In my estimates, the value of market production lost in Ireland was €229.7 million in 2015 and the difference between the results of the two studies is low regarding a 13–14 years divergence of the time settings. The methodological discrepancies seem to explain this difference as the Irish estimates are based on employment rates revised downward by only 2.25 percentage points, while here this adjustment is of greater magnitude (27.1 percentage points). When it comes to estimates from non-EU developed countries, the most recent American study reported indirect cost of suicide deaths as being $US 53.0 billion in 2013 [7], an equivalent of 0.32% of GDP. The share of GDP lost for this US study is more than double than the according share for Latvia–the country with the highest losses estimated here. However, the referred US estimates differ notably from mine in methodological terms, i.a. by including fringe benefits and household productivity losses while not accounting for lower productivity of those dying by suicide.

The major advantage of this research is the fact that it is the first study providing internationally comparable data on the production losses of suicide deaths, a major cost category of the total economic burden of suicides. Moreover, country-specific measures regarding labour market characteristics (real-life data on market entry and exit) and future economic growth are used and this reflects real differences among the countries investigated. With the use of homogenous data for these indicators, the peculiarities among the 28 states could not be unravelled.

On the other hand, the caveats of this analysis should also be pointed to. First, it only provides estimates on the burden of completed suicides in terms of potential production lost, while it does not investigate health care, medicolegal and intangible costs. However, the cost of mortality is a major cost driver of the suicide-attributable economic burden; therefore, my estimates provide useful insight into the topic. Second, although the model uses homogenous data from a set of countries, some of the measures employed do not represent the reality in a perfect manner; e.g. the employment rates of those suffering from depression possibly deviate from the real employment among suicide population. Yet, it seemed to be the best choice available to account for different employment engagement of suicide population in particular countries. Moreover, such potential inaccuracies resulting from the use of proxy input data have been addressed by the sensitivity analysis. Third, the use of the HCA for the assessment of production losses is criticized for inflating the real economic burden [22, 23]; still, it is the most common method for assessment of indirect cost and the alternative of the friction cost method has its own weaknesses [44]. The detailed discussion on pros and cons of the two methods is beyond the scope of this paper (and can be found elsewhere [24]) but the choice of HCA can be justified by the fact that also the previous studies used it and this allows for direct comparison between the cost estimates. Fourth, the estimates from this research might be biased downward because of under-reporting suicides [7, 45]; however, it is not clear how serious this issue is and under-reporting data is critically missing in international, comparable context [46]. Moreover, this is just one of the problems concerning validity and reliability of the suicide statistics. The cross-country study using WHO European mortality data shows that the quality of suicide statistics varies notably between countries while the reasons for this variability are numerous [47] making the magnitude of the figures’ uncertainty difficult to assess. All and all, until now the ambiguous quality of suicides statistics appears to be an unsolved problem [48] and the only reasonable way to account for this issue was to classify all the undetermined deaths as suicides in sensitivity analysis. However, the results appear not to be satisfactory in this scenario, because there is enormous by-country variation in this death cause category; e.g. there were only 2 deaths cases among men aged 10–69 years in Italy in 2015 while in Poland–which has lower population than Italy–the according figure was 1240. Finally, the analysis only accounts for the cost borne in formal economies and does not include the burden experienced in terms of household activities undone or unregistered production lost because of suicides. Unfortunately, with the data presently at hand, this shortcoming could not have been addressed.

## Conclusion

Because suicide is an avoidable mortality risk factor, public health actions aimed at prevention of suicides might not only reduce its health burden but also contribute to societies’ welfare. Therefore, preventive actions towards reduction of suicide incidence should be judged not only in terms of their cost but also benefits for production losses’ reduction resulting from lower mortality rates achieved.

## Supplementary Information

**Additional file 1.** Country-specific age groups distribution of production losses attributable to suicide mortality in European Union countries.

**Additional file 2.** Detailed, country-specific sensitivity analysis for estimates of production losses attributable to suicide deaths in European Union countries.

**Additional file 3.** Dataset.

## Data Availability

The dataset generated and analyzed during the current study is available in Additional file 3.

## Abbreviations

BS: Base scenario
CEE: Central and Eastern Europe
DR: Discount rate
EU: European Union
EU-28: 28 European Union states
GDP: Gross domestic product
GVA: Gross value added
HCA: Human capital approach
NE: Northern Europe
SE: Southern Europe
WE: Western Europe
YPPLL: Years of potential productive life lost

## Footnotes

1 For example, supposing that there were 85 suicide deaths among males aged 30–34, it was assumed that there were 17 deaths at each age in this interval.

2 For Denmark and Sweden data was not available and sex-specific average values for the remaining EU countries were used.

3 For 15–19 years of age employment rates I used 20–24 years employment rates because of low reliability and missing values in the former age interval for several countries (data for 4th quarter of 2015). This might bias the estimates upwards because employment rates are higher in the older age interval. Yet, this bias does not seem to have large impact on results as the number of suicides in the 15–19 age group is generally low. Moreover, in a majority of the countries the age of starting a first job is close to the upper bound of the age interval (19 years) and the employment rates at this age are plausibly closer to the rates for 20–24 years age interval.

4 Purchasing power parity of a particular country indicates how many units of national currency is needed in that country in order to maintain the purchasing power of one euro in the EU.

5 A ‘one-way’ sensitivity analysis refers to a single change of input parameter (e.g. discount rate) at a time with other parameters held constant. ‘Deterministic’ character of sensitivity analysis means that the isolated effects of changes in individual parameter is assessed, while ‘probabilistic’ sensitivity analysis assumes joint parameter uncertainty.

6 Gross value added is an alternative (to GDP) measure of productivity; the relationship between GDP and GVA is: GVA = GDP – Taxes on products + Subsidies on products. GVA is sometimes considered to be a superior to GDP in assessing economic welfare of population.

7 The minimal productivity was obtained by dividing minimum wage by average wage in particular economies; for details see the note of Table 3.
